# Identifying and Addressing Barriers and Facilitators for the Implementation of Internet of Things in Distributed Care: Protocol for a Case Study

**DOI:** 10.2196/44562

**Published:** 2023-09-28

**Authors:** Klas Palm, Carl Kronlid, Anders Brantnell, Marie Elf, Johan Borg

**Affiliations:** 1 Department of Civil and Industrial Engineering Uppsala University Uppsala Sweden; 2 School of Health and Social Sciences Dalarna University Falun Sweden

**Keywords:** barriers, digital health, facilitators, health care, implementation, internet of things, IoT solution, older people, person-centered care, technology

## Abstract

**Background:**

The internet of things (IoT) is recognized as a valuable approach to supporting health care to achieve quality and person-centered care. This study aims to identify the facilitators and barriers associated with implementing IoT solutions in health care within a Scandinavian context. It addresses the pressing need to adapt health care systems to the demographic changes occurring in Scandinavia. The vision of “Vision eHealth 2025,” a long-term strategic direction for digitalization in Sweden, serves as the background for this project. The implementation of IoT solutions is a crucial aspect of achieving the vision’s goal of making Sweden a global leader in using digitalization and eHealth opportunities by 2025. IoT is recognized as a valuable approach to supporting health care to achieve quality and person-centered care. Previous research has shown that there is a gap in our understanding of social and organizational challenges related to IoT and that the implementation and introduction of new technology in health care is often problematic.

**Objective:**

In this study, we will identify facilitating and hindering factors for the implementation of IoT solutions in social and health care.

**Methods:**

We will use an explorative design with a case study approach. The data collection will comprise questionnaires and qualitative interviews. Also, a literature review will be conducted at the start of the project. Thus, quantitative and qualitative data will be collected concurrently and integrated into a convergent mixed methods approach.

**Results:**

As of June 2023, data for the review and 22 interviews with the stakeholders have been performed. The co-design with stakeholders will be performed in the fall of 2023.

**Conclusions:**

This study represents a unique and innovative opportunity to gain new knowledge relevant and useful for future implementation of new technology at health care organizations so they can continue to offer high-quality, person-centered care. The outcomes of this research will contribute to a better understanding of the conditions necessary to implement and fully use the potential of IoT solutions. By developing cocreated implementation strategies, the study seeks to bridge the gap between theory and practice. Ultimately, this project aims to facilitate the adoption of IoT solutions in health care for promoting improved patient care and using technology to meet the evolving needs of health care.

**International Registered Report Identifier (IRRID):**

DERR1-10.2196/44562

## Introduction

### Overview

There is a growing movement toward distributed care, wherein health care services are increasingly offered in the home environment to foster a close and person-centered approach to health care delivery. In addition, an increasing number of older adults often live longer at home, even with cognitive and physical disabilities [1,2], and extensive support is usually required to maintain autonomy and independence in daily life [3]. The increase in the older population and their health care needs has put health care under extra pressure. For example, increased costs and a shift from the traditional single-disease paradigm to a more holistic patient-centered approach are considered among the greatest challenges for society in this century [4]. At the same time, personnel and financial constraints may reduce health care services or the quality of care for older adults. Due to this, there is a need to use technology to provide more efficient health care services. IT can support health care organizations in providing high-quality, person-centered care [5-7]. Information technology can reduce the burden on health care and its resources, enable better health care and cost savings, support more care at home, and thus reduce costs [8,9]. Examples of such technologies are the Internet of Things (IoT) and wearable technologies that could offer promising solutions for the care of older people. It also has the potential to improve the quality of life of older adults and allow them to maintain their independent lifestyles [10]. In addition, IoT is suggested to be crucial in transitioning care and rehabilitation from the hospital to care and rehabilitation at home [11]. IoT technology can also contribute to health promotion initiatives, reducing the need to provide treatment and hospitalization [12,13]. The implementation of IoT is a vital aspect of Sweden’s Vision eHealth 2025, which aims to make Sweden a global leader in digitalization and the opportunities provided by eHealth.

**Figure 1:**
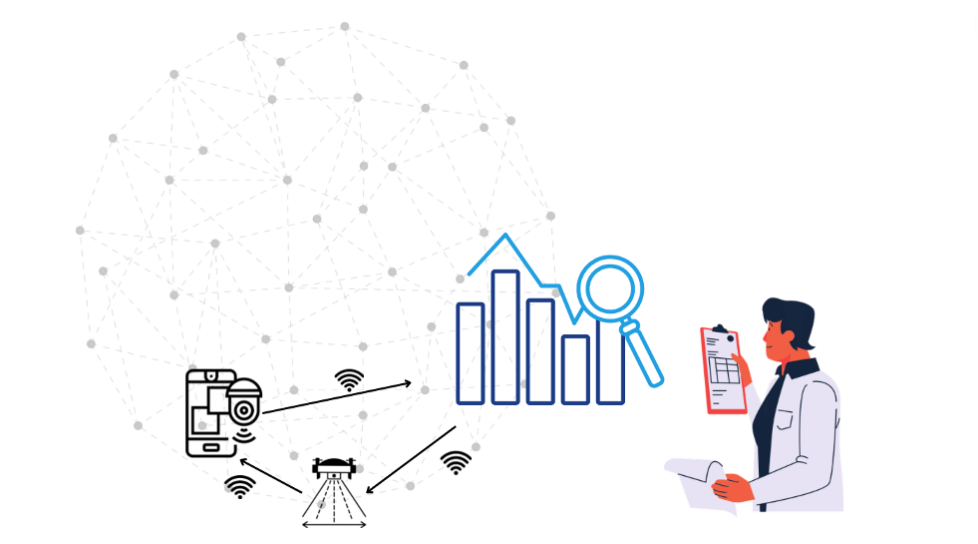
Illustration of how Internet of Things is defined in this study.

There is no unified definition of IoT, and many suggestions are put forth in the literature. One definition that is useful for our project is that IoT is “a network that connects uniquely identifiable devices (or things) to the internet, enabling them to collect, send, store, and receive data” [12]. From a technical point of view, an IoT architecture consists of several “layers.” Again, there is no unified agreement describing the IoT architecture, and the number of layers varies. For example, Nord et al [14] propose three layers: (1) sensing layer, (2) network layer, and (3) application layer, while Kashani et al [10] propose four layers: (1) perception layer, (2) network layer, (3) middleware layer, and (4) application or business layer. This means that in an IoT architecture, first, there is the “thing” that collects data; then, the data are sent somewhere via the internet; and lastly, the data are analyzed and used in or by some kind of application. IoT approaches can also be divided into the following five categories depending on their technological focus: (1) sensor-based IoT (eg, wearable sensors and environmental sensors), (2) resource-based IoT (eg, scheduling and resource allocation), (3) communication-based IoT (eg, technological approaches and algorithmic approaches), (4) application-based IoT (eg, monitoring systems and recommender systems), and (5) security-based IoT (eg, privacy, access control, and confidentiality). The definition of IoT in this research project is a network that connects uniquely identifiable sensor-based things to the internet, enabling them to collect, send, store, and receive data, as shown in Figure 1.

Previous research shows that most IoT-related research within the health care domain has a technological focus. Dantu et al [15] analyzed keywords for IoT research in the health care domain in a systematic literature review. They found 5 topic clusters: security and privacy, data, application, wireless network technologies, and cloud and smart health. The authors found that the social and organizational challenges (eg, factors related to users, health care professionals, and health care organizations) of IoT within health care have received little to no attention in existing research. They conclude that there is a gap in our understanding of social and organizational challenges related to IoT.

These are important perspectives when an IoT product is to be implemented in an ongoing business. Nord et al [14] have conducted a study of factors that influence IoT implementation. In that study, they state that theories related to IoT adoption and implementation appear to be nonexistent [14]. This can be troublesome when previous research has shown that implementing and introducing new technology in general in health care is often problematic [16,17]. However, there may be factors at several different levels that prevent or facilitate implementation. Flottorp et al [18] systematically reviewed factors that hinder and facilitate implementation in health care. They identified factors at seven different levels that influence implementation in health care: (1) innovation, (2) health care staff, (3) patients, (4) professional interaction, (5) incentives and economics, (6) capacity for organizational change, and (7) the surrounding society [18]. Venter et al [19] found several factors that hinder the implementation of welfare technology, such as (1) high costs, (2) lack of competence and knowledge of available solutions, (3) organizational issues, (4) resistance to change, (5) communication and cooperation across stakeholders, (6) legal complications, and (7) technological problems. As is visible in these studies, most of the factors influencing implementation are nontechnical. In line with this, Kashani et al [10] argue that one of the biggest challenges around IoT in health care right now is its implementation in the health care environment, thus underlining the need for research on these aspects.

Identifying and addressing factors that influence implementation is, however, only the first stage of actually implementing new solutions such as IoT and, through this, improving care. The factors will tell us what should be changed or addressed to achieve implementation. In order to make use of the factors, they need to be connected with relevant implementation strategies, that is, methods that target specific factors and aim to change these or underlying behaviors [20]. For instance, if health care practitioners do not use IoT solutions because they forgot, then a suitable implementation strategy would focus on forgetting and improving conditions to remind them to use the solution [21]. To our knowledge, studies on IoT have not examined or focused on implementation strategies. However, existing research on implementation in health care has identified a multitude of implementation strategies that fit with specific factors at the individual, organization, and system levels [20]. Technology-related factors do not have their own strategies as the technology is often given, allowing only slight local adjustments, and thus one should target other levels such as the individual’s interaction with the technology and the organizational fit of the technology. Still, on many occasions, connecting factors with suitable implementation strategies is rare [22], and the usual approach has long been to study factors and implementation strategies independently from each other, which implies that our knowledge of effective implementation strategies is incomplete [23].

Furthermore, we can state that a cocreation between researchers and concerned personnel in developing strategies for how new solutions should be implemented creates good conditions for the strategies to be well adapted and implemented [24]. We use this fact in this research project and intend, as a part of the project, to cocreate with staff to develop strategies based on the researched insights and the staff’s knowledge of the local context.

### Aim and Research Questions

The aim of the project is to contribute to an increased ability to use the potential of IoT solutions in society. In this study, we will identify facilitating and barrier factors for the implementation of IoT solutions in health care, including home care. To contribute to this aim, the study intends to answer the following two questions:

1. Research question 1: What are the barriers and facilitators when implementing IoT solutions within distributed care?
2. Research question 2: How to address barriers and facilitators when implementing IoT solutions within distributed care?

## Methods

### Design

**Figure 2:**
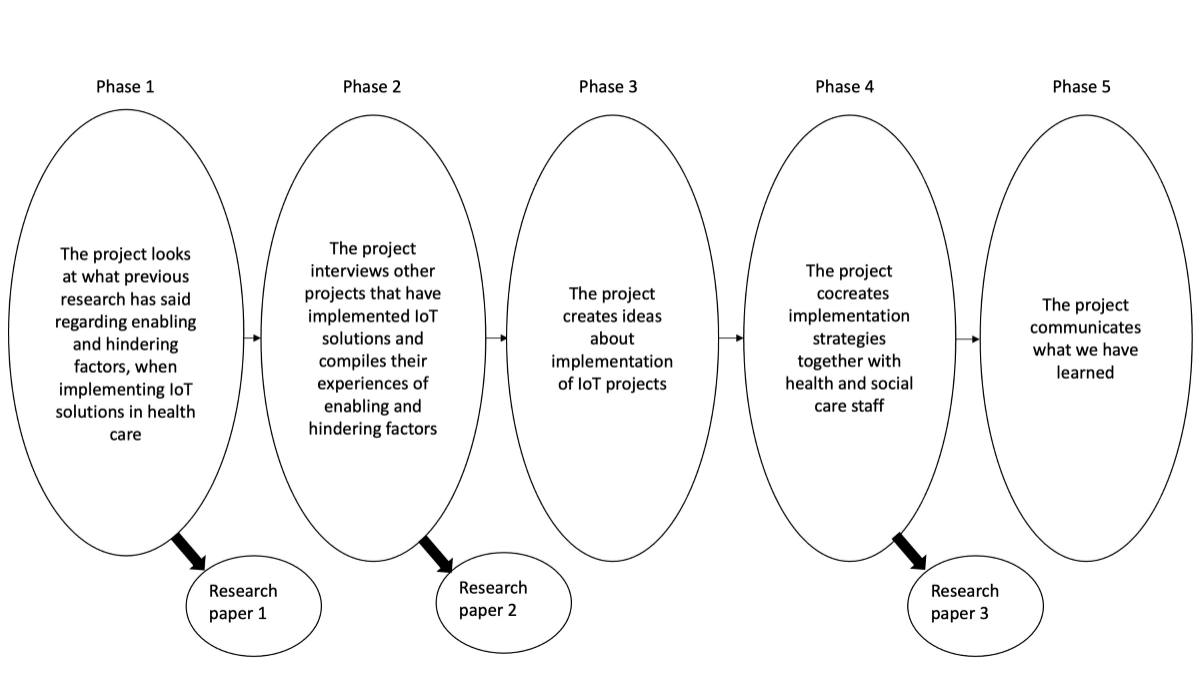
The 5 phases of the research process. IoT: internet of things.

The study will be performed in 5 phases (Figure 2). The aim of the first phase is to identify and categorize existing knowledge about barriers and facilitators for the implementation of IoT solutions in health care, including home care. The first phase will build on a systematic review. In the second phase, we will broaden the empirical base with more experiences from facilitating and barrier factors when implementing IoT solutions in Sweden. This will be done through a qualitative data collection from ongoing implementation projects of IoT solutions in care in Sweden. In the third phase, the project will analyze and draw conclusions from phases 1 and 2 and, based on this, formulate recommendations to strengthen facilitators and mitigate barrier factors when implementing IoT solutions in health care. In the fourth phase, these recommendations are to be applied in 2 implementation cases within the framework of ongoing safety-creating technology development. In this phase, there will also be a compilation of what we learned from these 2 implementation projects. In the fifth phase, the project will work on the use and dissemination of the generated results.

The knowledge generated by this project has the potential to benefit the fulfillment of Vision eHealth 2025. An important prerequisite, however, is that the knowledge generated by the project be communicated and used. Therefore, in phase 5, the researchers will collaborate with municipalities, regions, and the Swedish Association of Local Authorities and Regions to communicate results. Throughout the project, learning and experiences are shared through regular project meetings where new knowledge and experiences are shared between the project participants. Furthermore, new knowledge and experiences from a work package are applied and used in subsequent work packages. Experience is gained within the project’s parties by holding internal seminars and meetings where the project participants’ colleagues can take part in the experiences gained and the new knowledge that is developed within the project.

### Context

Many health care providers in Sweden realize that they face major demographic and resource challenges. To a certain extent, there is high confidence that digital solutions will help deal with these challenges. There is therefore relatively big support within municipalities and health care provider organizations for carrying out research promoting the implementation of IoT solutions. At the same time, several of the private companies that work on developing new IoT solutions perceive that there are major challenges in making use of their solutions. All in all, this means that the organizational context is expectant and generally positively disposed to this type of research project.

To run this research project, a research project committee has been created. The committee consists of researchers at the Department for Industrial Engineering and Management at Uppsala University; researchers at the School of Health and Welfare at Dalarna University, the Division of Assistive Technology in Council Regional Dalarna Healthcare Administration, Dalarna Science Park, and Falun’s and Rättvik’s municipalities; and a representative for producers of goods and services within IoT. The majority of these are also represented in a regional steering group for digitization and welfare technology in Dalarna.

### Data Collection

#### Data Collection to Respond to Research Question 1

We will conduct a systematic literature review to study what has already been written about implementing IoT technology. We will search for relevant articles in 4 databases: CINAHL, PubMed, Web of Science, and Scopus. A combination of Medical Subject Headings terms and keywords in titles and abstracts will be used, such as IoT and health care.

Data will also be collected from stakeholders’ experiences of implementation challenges, facilitators, and barriers in implementing IoT solutions in health care, including home care. Data are collected from ongoing projects on the implementation of IoT solutions. Empirical data are collected from IoT projects aimed at the implementation of IoT solutions in Sweden in health care, including home care. Interviews will be conducted with stakeholders involved in the implementation project. In total, it is estimated that between 20 and 30 respondents will be interviewed. Data collection takes place through semistructured interviews. The interviews are planned to take place digitally. Audio recording will be used if respondents accept this and then transcribed.

#### Data Collection to Respond to Research Question 2

To answer this question, conclusions will be drawn from research question 1. On the basis of these conclusions, support will be developed to create implementation strategies for IoT solutions within 2 pilot projects. These implementation strategies will be developed in cocreation with employed health and social care staff within the county Dalarna and municipalities in Dalarna within the 2 pilot projects. These pilot projects will be selected during the work on answering research question 1. The criteria for selecting pilot projects are that they must consist of projects offering health or social care and be ready to implement IoT-based solutions in accordance with this project’s definition of IoT. Data will be collected through a participative study in workshops for the development of implementation strategies and through interviews with representatives of key stakeholders involved in the implementation of the IoT solutions.

### Data Analysis

The following analysis method will be used to answer both research question 1 and research question 2.

Bibliometric and content analysis of previous literature will be used in the systematic literature review.

The analysis of barrier and facilitating factors from previous research will constitute the analytical framework in the research process, which means that the identified barrier and facilitating factors will be deductively categorized on the basis of existing research. Previous implementation research has generated a spectrum of perspectives that focus on barrier and facilitating factors for implementation, for example, Flottorp et al’s [18] previously identified seven different groups of barrier and facilitating factors: (1) guidelines, (2) care staff (eg, current knowledge), (3) patients (eg, preferences), (4) professional interaction, (5) incentives and finances (eg, remuneration), (6) capacity for organizational change (eg, mandate), and (7) society (eg, laws).

The categorization of the empirical data will be made on the basis of Flottorp et al’s [18] framework. The analysis also takes into account unique contextual conditions for implementation, which means that the analysis allows the identification of new factors and strategies that can complement previous research. The deductive approach is thus complemented by an inductive approach.

### Relation to Sustainable Development

The project contributes to Sustainable Development Goal (SDG) number 3 by increasing the ability to implement IoT-based technology, which creates better conditions for a continued or increased cost-effective health service to citizens. The project contributes to SDG number 5 by focusing on IoT solutions that create conditions for both men and women to enjoy good, individualized care. The project contributes to SDG number 9 by taking place in collaboration with industrial stakeholders working with developing products and services within IoT technology. The project is expected to contribute with an increased ability to develop products, test them, and implement solutions.

### Ethical Considerations

The Helsinki Declaration will be followed. The most overriding ethical issue in this type of research is of an integrity-infringing nature, as the researcher, during an interview, asks employees’ own thoughts about barriers and facilitators. Issues that may be discussed, such as fears, concerns, and difficulties related to barriers and facilitators, may be perceived as personal and private. The results will not be reported so that the respondents’ age, gender, profession, and other characteristics can function as identity markers. When presenting individual quotations, the research group will not use quotations where identity markers or other information are named or mentioned in such a way that the respondent risks being identified.

An application about the need for ethical review of the research has been submitted to the Swedish Ethical Review Authority. The authority has announced that ethical review is not necessary in this research, given that the questions do not regard any personal data.

## Results

As of June 2023, data for the review and 22 interviews with the stakeholders have been collected. The co-design with stakeholders will be performed in the fall of 2023. All data will be analyzed and disseminated during 2024.

## Discussion

### Overview

As one of the first studies to identify facilitators and barriers for implementing IoT solutions in health care in a Scandinavian context, we hope this project will contribute to better conditions to implement and thus use the potential of IoT solutions to contribute to the changes that need to take place in health care given the demographic changes we see in Scandinavia.

Sweden has a vision for digitalization in health care, Vision eHealth 2025. The vision sets out a long-term strategic direction for the digitalization area. It constitutes a joint collaboration platform between the Swedish Association of Local Authorities and Regions and the governmental stakeholders in the field. The vision is that by 2025, Sweden will be the best in the world at using the opportunities of digitalization and eHealth. Complementing the vision, there is also a national 3-year strategy for implementing it. The implementation is largely about developing new working methods and learning how new technology can be used operationally. In this context, implementing IoT solutions is an upcoming major issue.

Therefore, we hope that this research project will not just identify facilitators and barriers but also make use of those findings in cocreative workshops with health and social care staff in order to develop cocreated implementation strategies. The study will give insight into how knowledge from former implementation projects can be integrated into new implementation projects. Namely, we contribute knowledge about how a process for developing a strategy can be performed based on the experiences of other projects. In other words, we can also learn from what is easy and what is difficult in the transformation from gained insights into concrete actions in a new context.

### Strengths and Limitations

This project has a strong design with several phases that build on each other, validating and expanding the knowledge gained in previous phases. Additionally, the study includes 2 pilot cases in real-world health care settings, providing valuable insights into the implementation of IoT. While patients and older adults at home (end users) are important for understanding the full impact of IoT, they are not included in the sample as their perspectives are beyond this project’s scope. Future research needs will indeed include end users.

### Conclusions

This study represents a unique and innovative opportunity to gain new knowledge that is relevant and useful for implementing new technology in municipalities and health care organizations. By identifying and understanding the barrier and facilitating factors associated with implementing IoT solutions in health care, including care and service at home, the study can provide valuable insights into the challenges that need to be overcome and the strategies that can be used to facilitate successful implementation. The study can also help identify best practices and key success factors that can be used to guide future implementation efforts. Additionally, the study can contribute to developing person-centered care by using new technology in municipalities and health care organizations.

## Abbreviations

IoT: Internet of Things
SDG: Sustainable Development Goal

## Data Availability

The data sets generated during this study are available from the corresponding author on reasonable request.
